# Hengshun Aromatic Vinegar Ameliorates Vascular Endothelial Injury *via* Regulating PKCζ-Mediated Oxidative Stress and Apoptosis

**DOI:** 10.3389/fnut.2021.635232

**Published:** 2021-05-28

**Authors:** Xin Li, Meng Gao, Shenghu Zhu, Lianhong Yin, Bao Zhang, Yan Qi, Yanyan Zhao, Yongjian Yu, Lina Xu

**Affiliations:** ^1^Jiangsu Hengshun Vinegar Industry Co., Ltd., Zhenjiang, China; ^2^College of Pharmacy, Dalian Medical University, Dalian, China; ^3^School of Grain Science and Technology, Jiangsu University of Science and Technology, Zhenjiang, China

**Keywords:** vascular endothelial injury, hengshun aromatic vinegar, oxidative stress, apoptosis, PKCζ

## Abstract

Vascular endothelial injury (VEI) is an early event of atherosclerosis, and reversing endothelial dysfunction has become a new trend in the prevention and treatment of cardiovascular diseases. Hengshun aromatic vinegar (HSAV), a traditional vinegar, has been reported to have many pharmacological activities, but its effect against VEI and the molecular mechanism are still unknown. In this study, effects of HSAV on VEI were evaluated in H_2_O_2_-induced human umbilical vein endothelial cells (HUVECs) and methionine-induced VEI in rats. Results showed that HSAV significantly increased cell viability, inhibited apoptosis, and reduced the generation of reactive oxygen species (ROS) in H_2_O_2_-induced HUVECs. Meanwhile, HSAV decreased serum homocysteine (Hcy), endothelin 1 (ET-1), and oxidized low-density lipoprotein (ox-LDL) levels, increased nitric oxide (NO) and endothelin nitric oxide synthase (eNOS) levels, ameliorated pathological changes in rats with VEI induced by methionine. In parallel, HSAV relieved oxidative stress by decreasing malondialdehyde (MDA) level and increasing superoxide dismutase (SOD), glutathione (GSH), and glutathione peroxidase (GSH-Px) levels in rats with VEI. Mechanism studies indicated that HSAV markedly downregulated the expression of protein kinase C zeta (PKCζ), and consequently regulated sirtuin 1 (Sirt1)-mediated oxidative stress signal pathway, and protein inhibitor of activated STATy (PIASy)-mediated apoptosis pathway, leading to the alleviation of oxidative stress and inhibition of apoptosis. These regulative effects of HSAV were further validated by knockdown and overexpression of PKCζ *in vitro*. In conclusion, HSAV showed protective effect against VEI by inhibiting PKCζ and, thereby, ameliorating oxidative stress and inhibiting apoptosis. This study not only provides guidance for the consumption of vinegar in daily life but also promotes the development of diet supplement for disease prevention.

## Introduction

Coronary artery disease (CAD) is one of the major cardiovascular diseases, which has been indicated as the main leading cause of death globally (1). The risk factors of CAD development include genetic factors, environmental, and lifestyle. In recent years, mounting evidences suggest that the degree of stenosis is not significantly associated with clinical symptoms or acute coronary events (2, 3), which prompted the exploration of the underlying mechanism of CAD. Long-term studies have indicated that vascular endothelial injury (VEI) caused impairment of endothelial function is closely related to the occurrence and development of CAD (4–6). The abnormal vasomotor contraction, increased tension, platelet adhesion, aggregation, and thrombosis caused by VEI play initiating and promoting roles in the pathogenesis of CAD (7). Reversing endothelial dysfunction has become a new trend in prevention and treatment of CAD.

It has been widely recognized that oxidative stress and cell apoptosis are the main alterations related to VEI in CAD. Due to increased generation of oxygen free radicals, excessive reactive oxygen species (ROS) accumulate, which leads to the imbalance between the generation of ROS and antioxidant defense system (8). The capsular endothelial cells in vessel walls can produce many kinds of ROS (9). The oxidative stress during VEI has been proposed as a key initiating step in the pathogenesis of many vascular diseases, including atherosclerosis, vascular complications of diabetes hypercholesterolemia, which is also the principal cause of epigenetic changes that occur during aging (10, 11). Oxidative stress consequently induces inflammatory injury and endothelial apoptosis, which ultimately lead to VEI (12). As such, oxidative stress can induce apoptosis, which further aggravate oxidative stress during VEI. Understanding oxidative stress and endothelial apoptotic signal pathways that are altered in VEI may enable greater understanding the pathogenesis and foster the development of therapeutic drugs for CAD.

Protein kinase C (PKC) is widely distributed in mammalian organs, tissues, and cells, which plays important role in regulating cell metabolism, growth, proliferation, and differentiation (13). The downregulation of PKCζ can stabilize the intestinal cytoskeleton, enhance cellular microtubule stability and barrier function, and prevent oxidative damage to the intestinal barrier (14). Inhibition of PKCζ protected cell injury, and modulating PKCζ pathway prevented high glucose-induced oxidative stress in vascular smooth muscle cells (15). Indomethacin, a non-steroidal anti-inflammatory drug, activates the PKCζ pathway, leading to apoptosis and disruption the physiological balance of mitochondrial dynamics by promoting mitochondrial overdivision and dysfunction (16). We also found that PKCζ expression was dramatically upregulated in the injured endothelial cells. Hence, PKCζ may be a potential target for the development of anti-VEI drugs through relieving oxidative stress and inhibiting cell apoptosis.

Over the past few decades, preventive and therapeutic strategies have substantially improved the prognosis of patients suffering from CAD. However, the risk of such disease is still high, and only a few patients can prevent its progression by medication that may cause a series of adverse reactions including gastrointestinal bleeding and liver damage (17, 18). Recently, nutraceutical approach has been proposed to counteract the increasing burden of CAD. It has been indicated that consumption of foods rich in antioxidant activity is associated with a reduced risk of cardiovascular events (19).

Hengshun aromatic vinegar (HSAV) is a kind of fermented acidic liquid food, which has been used as condiment for hundreds of years in China. It was produced by solid layered fermentation process with high-quality glutinous rice as raw material and special wheat as saccharifying starter (20). HSAV is rich in a variety of antioxidant components, such as polyphenols, flavonoids, and melanosine (21). These antioxidant components can scavenge oxygen free radicals and reduce the level of cellular oxidative stress, which play roles in lowering blood lipid, prevent atherosclerosis, and are beneficial to human health (22). Meanwhile, HSAV also contains a variety of free amino acids, some of which have strong antioxidant capacity, including histidine, methionine, cysteine, tryptophan, tyrosine, and so on (21). Our previous studies found that HSAV showed protective effects on non-alcoholic fatty liver disease (NAFLD) through reducing inflammation and lipogenesis (22). Remarkably, the malondialdehyde (MDA) level was descended by HSAV treatment in rats with NAFLD. However, effect of HSAV on oxidative stress in VEI remains unknown. In this study, we evaluated the protective effect of HSAV on VEI in H_2_O_2_-induced human umbilical vein endothelial cells (HUVECs) and high methionine-induced rats, and the potential molecular mechanisms of HSAV were further elucidated.

## Methods

### Chemicals and Materials

HSAV was obtained from Jiangsu Hengshun Vinegar Industry Co., Ltd (Zhenjiang, China). Dulbecco's modified Eagle's medium (DMEM) and fetal bovine serum (FBS) were purchased from Gibco (California, USA). Cell Counting Kit (CCK-8) was purchased from Seven Biotech Co., Ltd. (Beijing, China). Rat homocysteine (Hcy) Elisa Kit was obtained from CUSABIO (Texas, USA). Rat endothelin 1 (ET-1), endothelin nitric oxide synthase (eNOS), nitric oxide (NO), superoxide dismutase (SOD), MDA, glutathione (GSH), glutathione peroxidase (GSH-Px), and oxidized low-density lipoprotein (ox-LDL) assay kits were purchased from Nanjing Jiancheng Institute of Biotechnology (Nanjing, China). Tissue protein extraction kit was purchased from KEYGEN Biotech (Nanjing, China) and enhanced bicinchoninic acid (BCA) protein assay kit was purchased from Beyotime Institute of Biotechnology (Jiangsu, China). *TransDetect In Situ* fluorescein TUNEL cell apoptosis detection kit, protein marker, was purchased from TransGen Biotech (Beijing, China). The primary antibody of PKCζ was purchased from Bioss (BeiJing, China), and Sirtuin 1 (Sirt1) was purchased from Cell Signaling Technology (Massachusetts, USA). The primary antibodies of nuclear factor E2-related factor 2 (Nrf2), Kelch-like ECH-associated protein 1 (Keap1), glutathione S-transferase (GST), NAD(P)H quinone dehydrogenase 1 (NQO1), protein inhibitor of activated STATy (PIASy), cellular tumor antigen p53 (p53), Caspas9, Caspas3, Bcl2-associated X (BAX), Bcl2 apoptosis regulator (Bcl2), and glyceraldehyde-3-phosphate dehydrogenase (GAPDH) were purchased from Proteintech Group (Chicago, USA). PKCζ siRNA (sense: 5′-CCAAAUUUACGCCAUGAAATT-3′, antisense: 5′-UUUCAUGGCGUAAAUUUGGTT-3′) and PKCζ overexpression plasmids were designed and produced by GenePharma (Shanghai, China). Lipofectamine2000 reagent was purchased from Thermo Fisher Scientific (Waltham, America).

### Cell Culture and Cytotoxicity of Hengshun Aromatic Vinegar in Human Umbilical Vein Endothelial Cells

HUVECs were purchased from the Institute of Biochemistry Cell Biology (Shanghai, China) and incubated with DMEM containing 10% FBS. To evaluate the cytotoxicity of HSAV, HUVECs were inoculated in 96-well-plates at a concentration of 5 × 10^3^/ml overnight. HSAV was diluted with DMEM and added to the cells at different concentrations (0.25, 0.375, 0.5, 0.75, 1, 1.5, 2, 2.5, and 3%, *v/v*). After incubating for 12 and 24 h, the cytotoxicity of the HSAV in HUVECs was assayed by CCK-8 method (*n* = 6). After treatment, the culture medium was sucked out and 10% CCK-8 reagent was added and incubated at 37°C for 1 h. The absorbance value was measured at 450 nm and the cell viabilities were calculated according to the absorbance value.

### Model Induction and Hengshun Aromatic Vinegar Treatment in Human Umbilical Vein Endothelial Cells

HUVECs were seeded into 96-well-plates at a concentration of 5 × 10^3^/ml overnight. To optimize suitable molding conditions, HUVECs were treated with H_2_O_2_ solutions (50, 100, 200, 400, and 800 μmol/L) for 1, 2, and 4 h, respectively. Cell viabilities were detected by CCK-8 method. In order to evaluate the effect of HSAV on injured HUVECs, cells were treated with HSAV at concentrations of 0.375, 0.75, and 1.5% (*v*/*v*) for 24 h, and then incubated with H_2_O_2_ (200 μmol/L) for 2 h (*n* = 6). Finally, cell viability was determined by CCK-8 method. In addition, cell morphology was imaged using a phase contrast microscope (Nikon, Japan).

### Reactive Oxygen Species Level in Human Umbilical Vein Endothelial Cells

After HSAV and H_2_O_2_ intervention, HUVECs were treated and incubated with DCFH-DA probe (10 μmol/L) for 20 min. Then, cells were washed with serum-free medium three times to remove the residual DCFH-DA probe. The ROS fluorescence was observed under a fluorescence microscopy (Olympus, Tokyo, Japan) with 200× magnification.

### Animals and Experimental Design

Male SD rats weighing 180–200 g were obtained from Liaoning Changsheng Biotechnology Co., Ltd [SCXK (Liao) 2015-0001, Benxi, China]. The rats were fed under the conditions of temperature (21 ± 3°C) and humidity (60 ± 5%), with free access to food and water. All animal care was according to the Laboratory Animal Care and Use Guideline provided by the National Institutes of Health and the treatment protocols were approved by the Institutional Ethics Committee of Dalian Medical University.

After a week of acclimatization, rats were randomly divided into seven groups (*n* = 8), including control, vinegar control (1.250 ml/kg), model, low-dose of HSAV (0.625 ml/kg), middle-dose of HSAV (0.833 ml/kg), high-dose of HSAV (1.250 ml/kg) groups, and positive control group, respectively. The rats in control group and vinegar control group were fed with normal diet, while rats in the other groups were given high-methionine diet (containing 3% methionine in normal diet). Meanwhile, rats in vinegar control, low dose, middle dose, and high dose of HSAV groups were administered by corresponding dose of HSAV (diluted with purified water). Rats in positive drug group were intragastrically given positive control drug (consisting of 1 mg/kg folic acid, 2 mg/kg vitamin B2, and 10 μg/kg vitamin B12), while rats in control and model group were given distilled water. Corresponding drugs were intragastrically administrated to rats once a day (10 ml/kg) for consecutive 8 weeks. After the last dose of drug administration, rats were fasted for 12 h, and serum and thoracic aorta samples were promptly collected for the following tests.

### Assessment of Biochemical Parameters in Rats

The serum levels of Hcy, ET-1, eNOS, and ox-LDL were estimated using ELISA kits, and the serum levels of NO, SOD, MDA, GSH, and GSH-Px were examined using assay kits according to the manufacturer's instructions.

### Histopathological Assay

The thoracic aorta tissues were rapidly removed after sacrifice, preserved in 10% neutral buffered formalin, and embedded in paraffin. The tissue paraffin block was cut into paraffin sections with a thickness of 4 μm, stained with hematoxylin and eosin (HE), and observed by Eclipse TE2000-U microscope (Nikon, Japan) at 100× and 400× magnifications. The average histological score of each group was calculated, and the degree of lesion was graded from zero to four depending on severity: no lesion (0), minimal (1), mild (2), moderate (3), and severe (4).

### TUNEL Assay

HUVECs were fixed with 10% formaldehyde for 20 min after treatment, and propagated by 0.5% Triton-100 for 10 min. Then, cells were incubated with TUNEL reaction mixture (50 μl TdT + 450 μl fluorescein-labeled dUTP). TUNEL staining of paraffin tissue sections was also performed according to the instruction of TUNEL detection Kit. Then, the fluorescence intensity of HUVECs and tissue sections were observed by a fluorescence microscopy (Olympus, Tokyo, Japan) with 200× magnification.

### Immunofluorescence Assay

The paraffin sections of thoracic aorta were dewaxed with xylene and rehydrated with gradient alcohol. The sections were treated with sodium citrate antigen repair solution in boiling water for 20 min. Then, the sections were incubated with PKCζ primary antibody at 4°C in a humidified box. On the next day, they were incubated with an Alexa fluorescein-labeled secondary antibody out of light for 1 h, and then stained with 1.0 μg/ml 4′,6-diamidino-2-phenylindole (DAPI) for 10 min. After washing with PBS, the images were obtained by a fluorescence microscopy (Olympus, Tokyo, Japan) with 200× magnification.

### Western Blotting Assay

Total protein samples were extracted from cells and tissues and quantified by BCA kits. The denatured protein was loaded and separated on appropriate proportion of SDS-PAGE gel (8–12%), transferred onto PVDF membranes, and then blocked with 5% non-fat milk. Then, the membranes were incubated with the primary antibodies at 4°C overnight. On the following day, the membranes were incubated with HRP-conjugated goat anti-rabbit secondary antibody. Then, the protein expression was detected by the enhanced chemiluminescence (ECL) method, and imaged with a ChemiDoc^™^XRS Imaging System (Bio-Rad Laboratories). The relative expression of target protein was normalized to that of GAPDH.

### Silencing of Protein Kinase C Zeta siRNA Transfection in Human Umbilical Vein Endothelial Cells

HUVECs were cultured in six-well-plates overnight to achieve 60–70% confluence, and transfected with PKCζ siRNA or negative control (NC) siRNA using Lipofectamine 2000 for 12 h. Then, cells were exposed to HSAV (1.5%) for 24 h and treated with H_2_O_2_ (200 μmol/L) for an additional 2 h. After such treatment, cell viability and the level of intracellular ROS of HUVECs were detected. The expression levels of PKCζ, Sirt1, Keap1, PIASy, and P53 were evaluated by Western blotting.

### Overexpression of Protein Kinase C Zeta in Human Umbilical Vein Endothelial Cells

The PKCζ overexpression plasmid and empty vector plasmid (NC) were transfected into the HUVECs cells using Lipofectamine 2000 for 24 h. After treatment with HSAV and H_2_O_2_, cell viability, intracellular ROS, and expression levels of PKCζ, Sirt1, Keap1, PIASy, and P53 in HUVECs were detected.

### Statistical Analysis

The experimental data were expressed as mean ± standard deviation (SD). GraphaPad Prism 5.0 Software (CA, USA) was used for data analysis. Significant differences among multiple groups were analyzed by one-way ANOVA test followed by Newman–Keuls test and the unpaired *t*-test was carried out when comparing two different groups.

## Results

### Effect of Hengshun Aromatic Vinegar on H_2_O_2_-Induced Injury in Human Umbilical Vein Endothelial Cells

**Figure 1:**
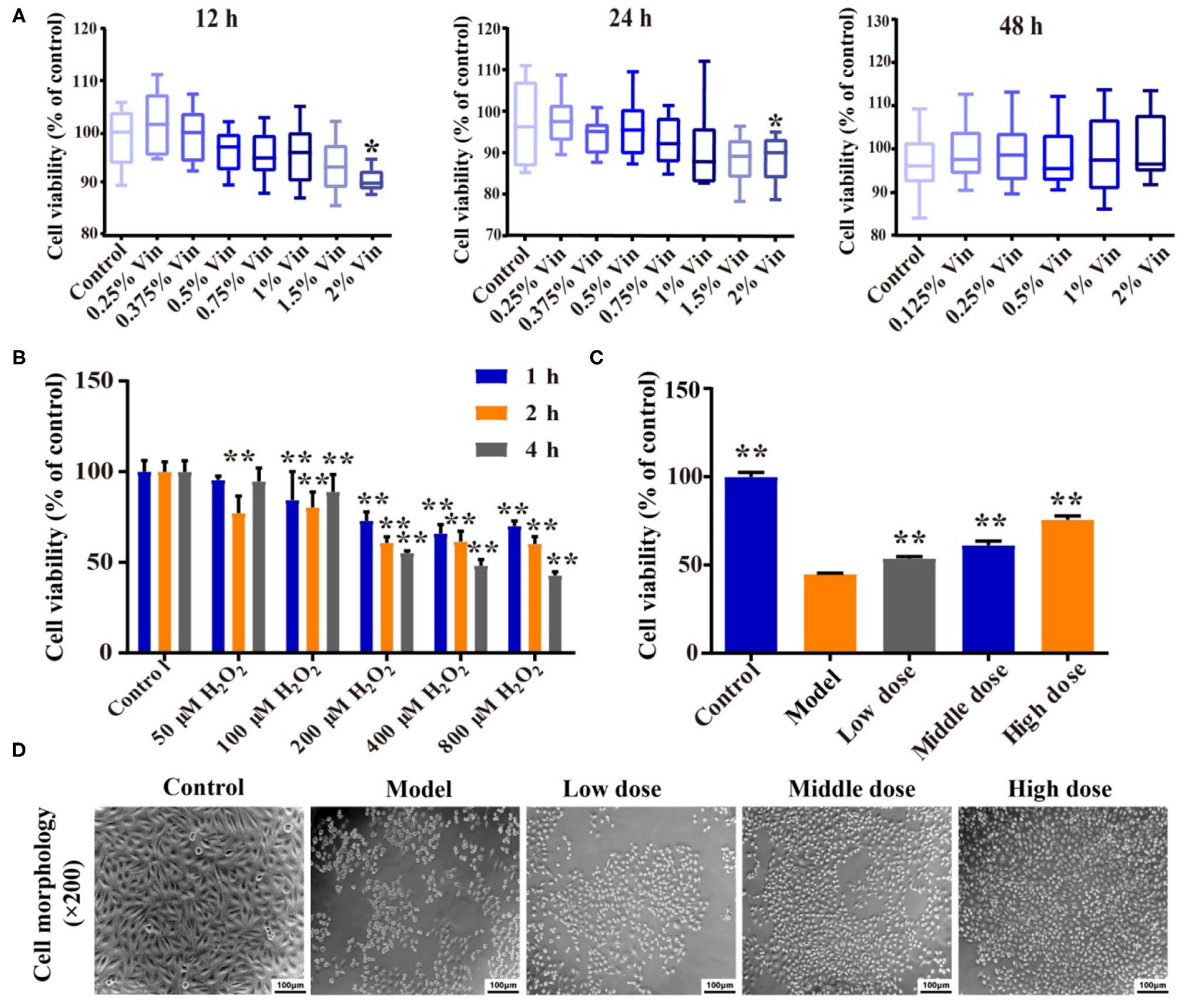
Hengshun aromatic vinegar (HSAV) relieves H_2_O_2_-induced human umbilical vein endothelial cells (HUVECs) injury. **(A)** Cell viabilities of HUVECs treated with different concentrations of HSAV for 12, 24, and 48 h by CCK-8 method (*n* = 6). Data are presented as the mean ± SD. ^*^p < 0.05, compared with the control group. **(B)** Cytotoxicity of H_2_O_2_ on HUVECs (*n* = 6). ^**^p < 0.01, compared with the control group. **(C)** Effect of HSAV on cell viability of HUVECs induced by H_2_O_2_ (*n* = 6). Data are presented as the mean ± SD. ^**^p < 0.01, compared with the model group. **(D)** Effects of HSAV on cell morphology of H_2_O_2_-induced HUVEC (*n* = 3).

As shown in Figure 1A, HSAV with the concentration no higher than 1.5% had no cytotoxicity on HUVECs for 12, 24, and 48 h. Therefore, 0.375, 0.75, and 1.5% HSAV were selected as low, middle, and high dose to treat HUVECs for 24 h in the following study. Then, the cell modeling conditions were optimized by evaluating the inhibitory effect of different concentrations (50, 100, 200, 400, and 800 μmol/L) of H_2_O_2_ on the viability of HUVECs at 1, 2, and 4 h, respectively. Results indicated that the cell viabilities of HUVECs were significantly decreased after H_2_O_2_ treatment for 2 and 4 h (Figure 1B), which was significantly decreased to 60.8% when HUVECs were treated with 200 μmol/L H_2_O_2_ for 2 h (Figure 1B). Therefore, treatment with 200 μmol/L H_2_O_2_ for 2 h was selected to establish injury model in HUVECs. Then, we evaluated the effect of HSAV on HUVECs induced by H_2_O_2_. Compared with model group, the cell viability was significantly elevated in HSAV treatment groups and showed a concentration-dependent trend (Figure 1C). At the same time, the cell morphology gradually reappeared to normal with HSAV treatment (Figure 1D). These results indicated that HSAV alleviated the injury of HUVECs induced by H_2_O_2_.

### Effects of Hengshun Aromatic Vinegar on Vasoactive Substances in Methionine-Induced Rats

**Figure 2:**
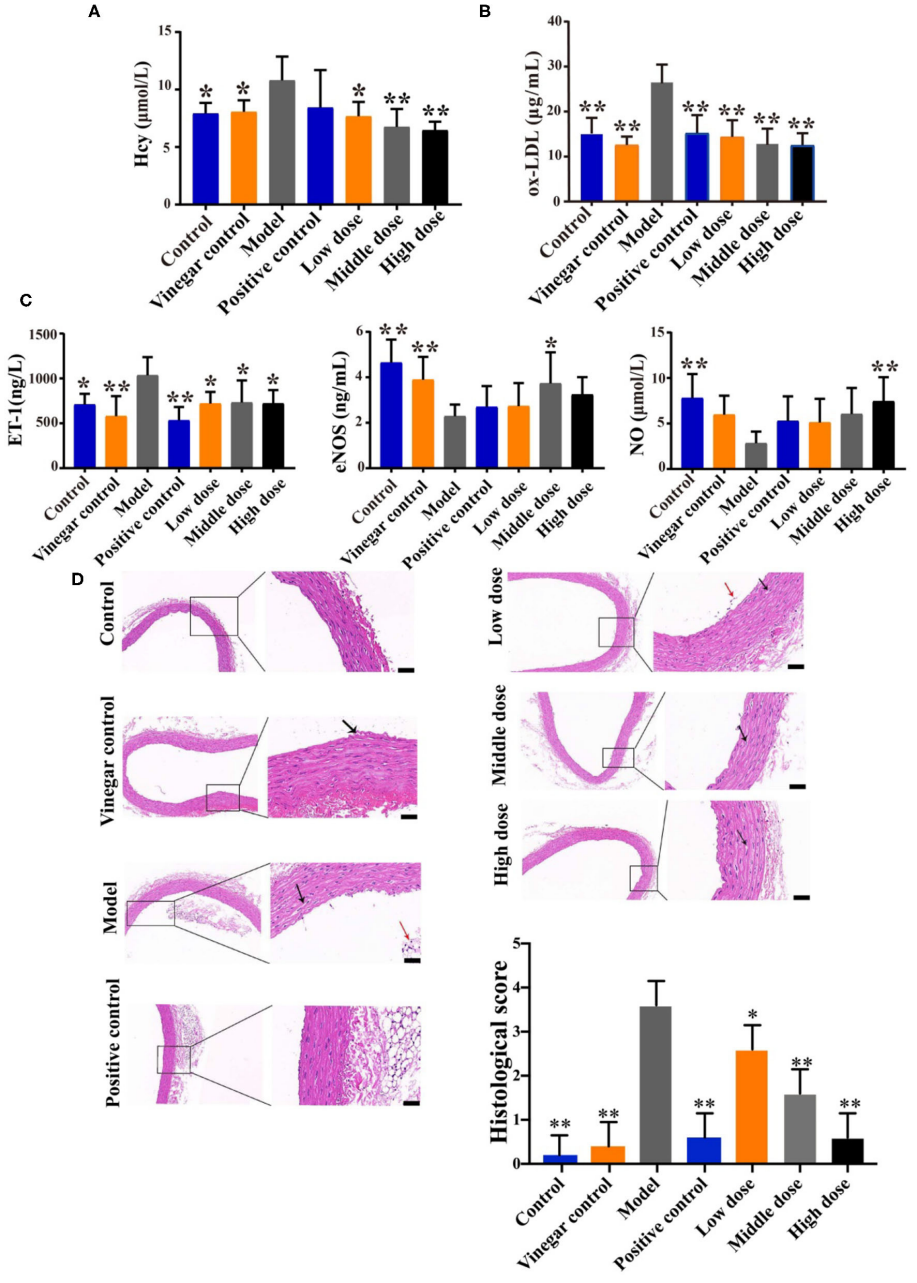
HSAV ameliorates vascular endothelial injury in methionine induced rats. **(A)** Homocysteine (Hcy) level; **(B)** oxidized low-density lipoprotein (ox-LDL) level; **(C)** levels of endothelin 1 (ET-1), nitric oxide (NO), and endothelin nitric oxide synthase (eNOS) in serum (*n* = 8); **(D)** HE staining of thoracic aorta (100×, 400×) (*n* = 3). Scale bar = 100 μm. Data are presented as the mean ± SD. ^*^p < 0.05, ^**^p < 0.01 compared with the model group.

Compared with the control group, serum level of Hcy in the model group was significantly increased (*p* < 0.05), while HSAV treatment can significantly decrease Hcy level compared with the model group (*p* < 0.05, Figure 2A). LDL oxidation is a key step in the formation of atherosclerotic plaques. Figure 2B displays that ox-LDL level in the model group was significantly higher than that of the control group, whereas it was significantly decreased in HSAV treatment group compared with the model group. The decreased ox-LDL level suggested that HSAV can reduce the risk of atherosclerosis. Moreover, ET-1 level was increased, and serum levels of NO and eNOS were decreased in the model group (*p* < 0.05, Figure 2C) compared with the control group, while HSAV markedly reversed the abnormal changes in the levels of ET-1, NO, and eNOS caused by H_2_O_2_ treatment (*p* < 0.05, Figure 2C). It revealed that HSAV regulated the secretion of vasoactive substances including vasoconstrictive factor ET-1, vasorelaxing factor NO and eNOS, which can relieve injury in HUVECs.

### Effect of Hengshun Aromatic Vinegar on the Pathological Changes in Methionine-Induced Rats

To evaluate the effect of HSAV on VEI *in vivo*, the rat VEI model was established with high-methionine diet. HE staining (Figure 2D) of thoracic aorta showed that several vascular smooth cells showed moderately edema and loose cytoplasm (black arrows) in model group. Furthermore, severe loss of endothelial cells and more endothelial cells shed in the vascular lumen (red arrows) were observed in model group. High dose of HASV and positive drug significantly improved the edema and loose cytoplasm, and reversed the changes of vascular endothelial structures. The histological score was significantly decreased in HSAV treatment groups (Figure 2D).

### Effects of Hengshun Aromatic Vinegar on Oxidative Stress *in vitro* and *in vivo*

**Figure 3:**
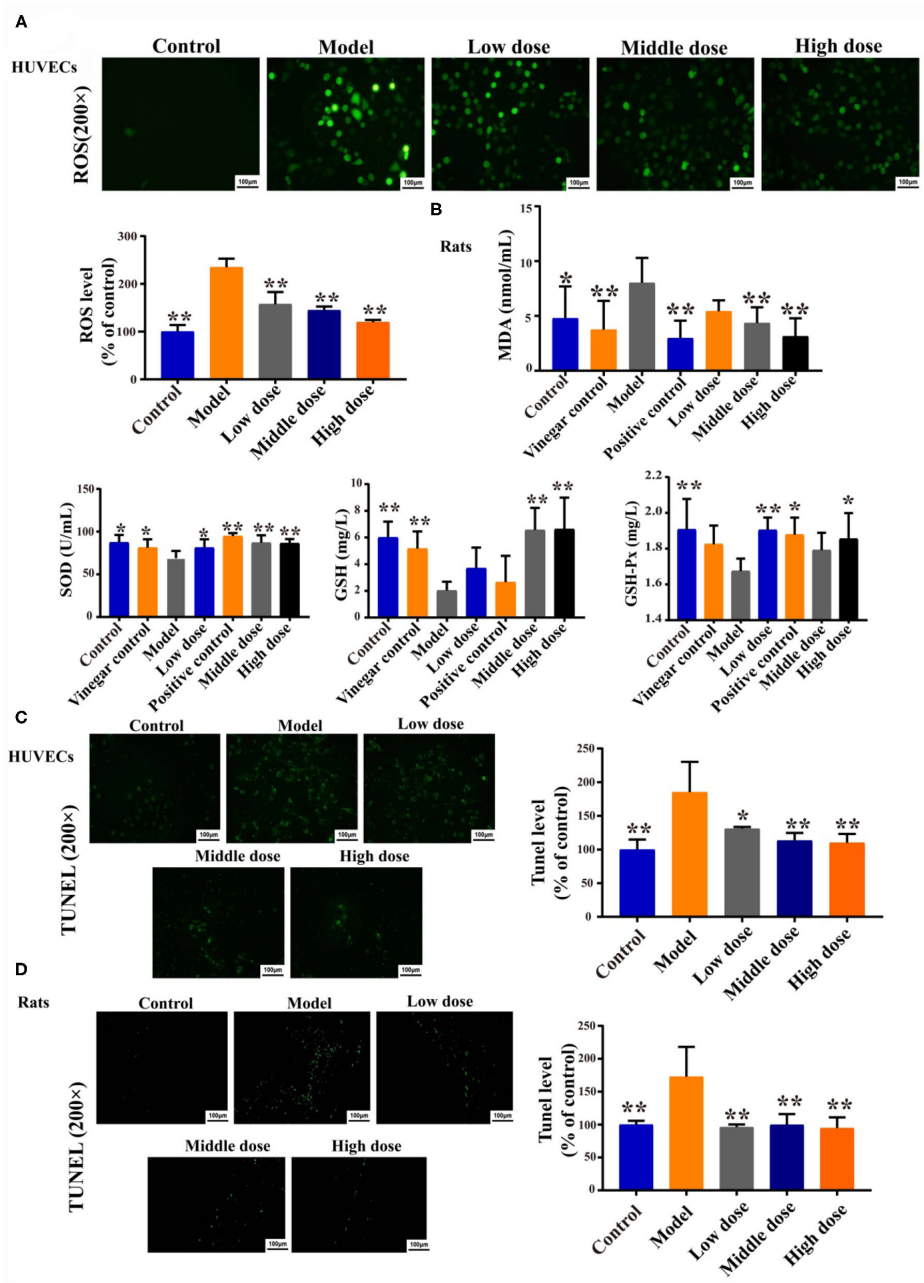
HSAV ameliorates oxidative damage and apoptosis *in vitro* and *in vivo*. **(A)** The cellular reactive oxygen species (ROS) in HUVECs (*n* = 3); **(B)** Malondialdehyde (MDA), superoxide dismutase (SOD), glutathione (GSH), and glutathione peroxidase (GSH-Px) levels in rat serum (*n* = 8); **(C)** TUNEL assay of HUVECs (*n* = 3); **(D)** TUNEL assay of thoracic aorta (*n* = 3). Data are presented as the mean ± SD. ^*^p < 0.05, ^**^p < 0.01, compared with the model group.

The fluorescence intensity of ROS in HUVECs significantly increased in model group compared with control group (Figure 3A). However, HSAV markedly decreased the fluorescence intensity of ROS compared with model group (Figure 3A). In addition, HSAV significantly increased the serum levels of SOD, GSH, and GSH-Px, and declined MDA level compared with model group (Figure 3B).

### Effect of Hengshun Aromatic Vinegar on Apoptosis *in vitro* and *in vivo*

Results of the TUNEL assay revealed that H_2_O_2_ inducement caused an increase of apoptosis in HUVECs, whereas the apoptotic cells were decreased compared with the model group when HUVECs were treated with HSAV (Figure 3C). Consistently, apoptosis cells of thoracic aorta were also decreased by HSAV treatment compared with model group (Figure 3D). These findings implied that HSAV effectively inhibited cell apoptosis of VEI *in vitro* and *in vivo*.

### Effect of Hengshun Aromatic Vinegar on the Expression of Protein Kinase C Zeta

**Figure 4:**
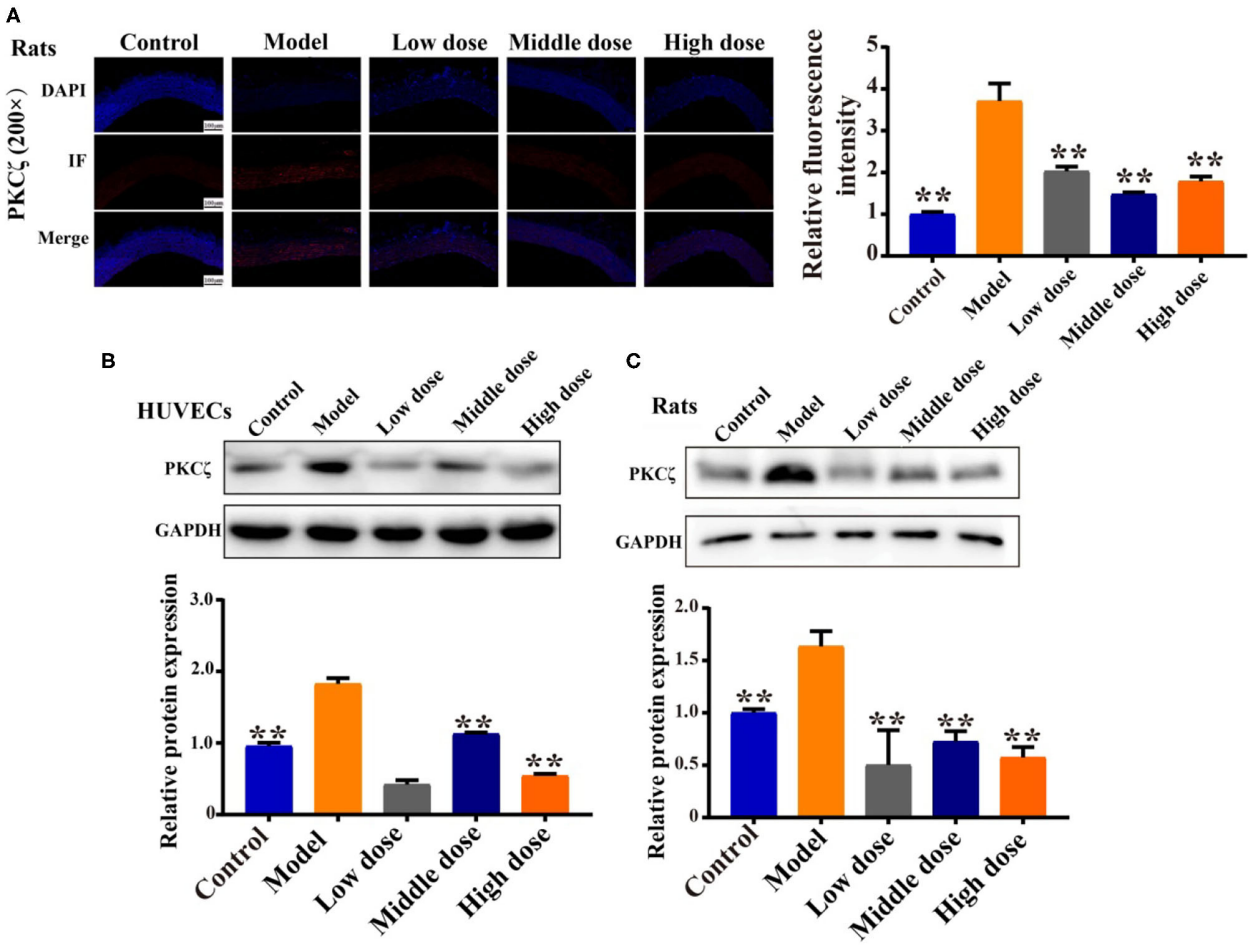
HSAV reduces protein kinase C zeta (PKCζ) protein expression *in vitro* and *in vivo*. **(A)** The expression of PKCζ in rats thoracic aorta by immunofluorescence (200×) (*n* = 3). **(B)** The expression of PKCζ in HUVECs by Western blot assay (*n* = 3). **(C)** The expression of PKCζ in rat by Western blot assay (*n* = 3). Data are presented as the mean ± SD. ^**^p < 0.01, compared with the model group.

Immunofluorescence results show that the fluorescence intensity of PKCζ in aorta tissue was significantly enhanced in the model group compared with the control group, while it was significantly decreased by HSAV treatment (Figure 4A). Meanwhile, H_2_O_2_ inducement significantly increased PKCζ expression in HUVECs compared with control group, while HSAV decreased expression level of PKCζ by Western blotting assay (Figure 4B). Consistently, the expression level of PKCζ in aorta tissues was also significantly downregulated by HSAV compared with the model group (Figure 4C), which was consistent with the results of immunofluorescence.

### Effect of Hengshun Aromatic Vinegar on the Expression of Proteins Related to Oxidative Stress Pathway

**Figure 5:**
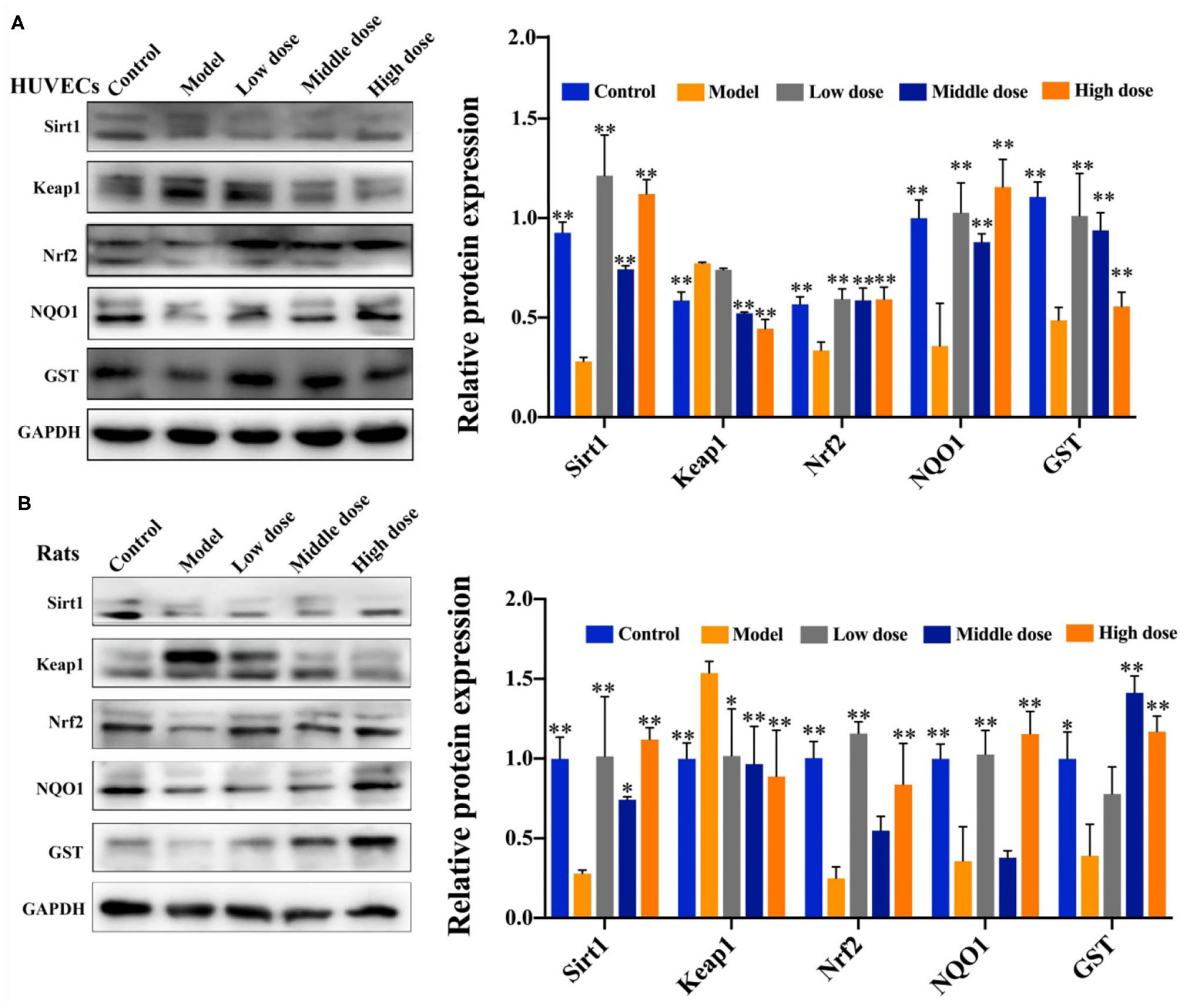
HSAV adjusts PKCζ-mediated oxidative stress signal pathway *in vitro* and *in vivo*. The expression levels of sirtuin 1 (Sirt1), Ketch-like ECH-associated protein 1 (Keap1), nuclear factor E2-related factor 2 (Nrf2), NAD(P)H quinone dehydrogenase 1 (NQO1), and glutathione 5-transferase (GST) in HUVECs **(A)** and rat thoracic aorta **(B)** by Western blot assay (*n* = 3). Data are presented as the mean ± SD. ^*^p < 0.05, ^**^p < 0.01, compared with the model group.

Results in Figure 5A show that, compared with the control group, the expression levels of Sirt1, Nrf2, NQO1, and GST in HUVECs were significantly decreased, and the expression of Keap1 was remarkably upregulated in model group. Compared with model group, HSAV significantly elevated the expression levels of Sirt1, Nrf2, NQO1, and GST, and decreased the expression of Keap1 in HUVECs (Figure 5A). In addition, effects of HSAV on proteins associated with oxidative stress pathway in rats were consistent with that of HUVECs (Figure 5B). These results demonstrated that HSAV regulated the downstream oxidative stress pathway of PKCζ in alleviatingendothelial injury.

### Effect of Hengshun Aromatic Vinegar on the Expression of Proteins Associated With Apoptosis Pathway

**Figure 6:**
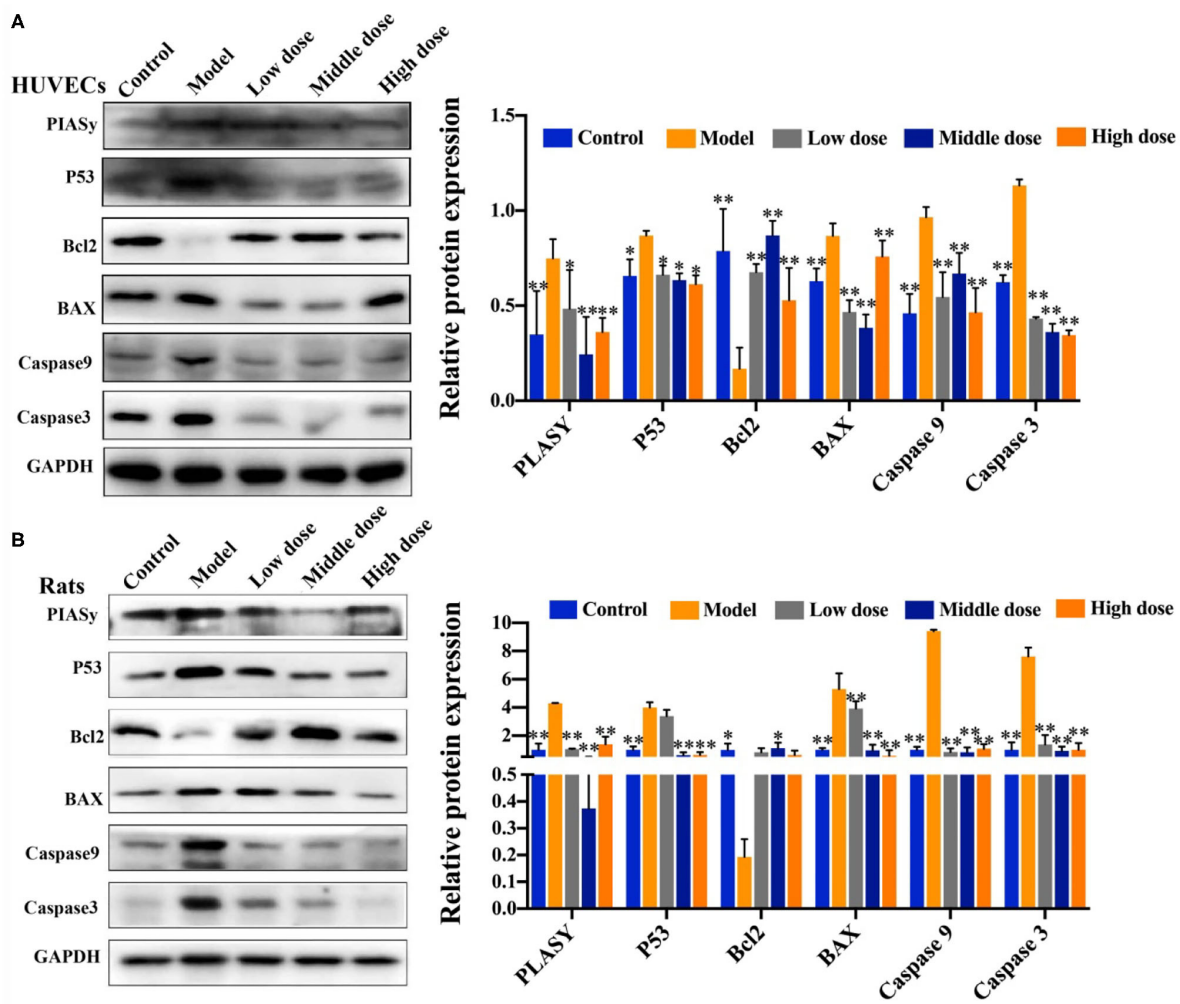
HSAV adjusts PKCζ-mediated apoptosis signal pathway *in vitro* and *in vivo*. The expression levels of protein inhibitor of activated STATy (PIASy), cellular tumor antigen p53 (P53), Bcl2 apoptosis regulator (Bcl2), Bcl2-associated X (BAX), Caspase3, and Caspase9 in HUVECs **(A)** and rat thoracic aorta **(B)** by Western blot assay (*n* = 3). Data are presented as the mean ± SD.^*^p < 0.05, ^**^p < 0.01, compared with the model group.

As shown in Figure 6, compared with the control group, Bcl2 expression was significantly downregulated, and expression levels of PIASy, P53, BAX, Caspase3, and Caspase9 were significantly upregulated in the model group. Of note, compared with the model group, HSAV markedly increased the expression of Bcl2, and decreased the expression levels of PIASy, P53, BAX, Caspase3, and Caspase9 (Figure 6). These results revealed that HSAV regulated the downstream apoptotic pathway of PKCζ in alleviating endothelial injury.

### Effect of Hengshun Aromatic Vinegar on Oxidative Stress and Apoptosis in Human Umbilical Vein Endothelial Cells With Protein Kinase C Zeta Silencing

**Figure 7:**
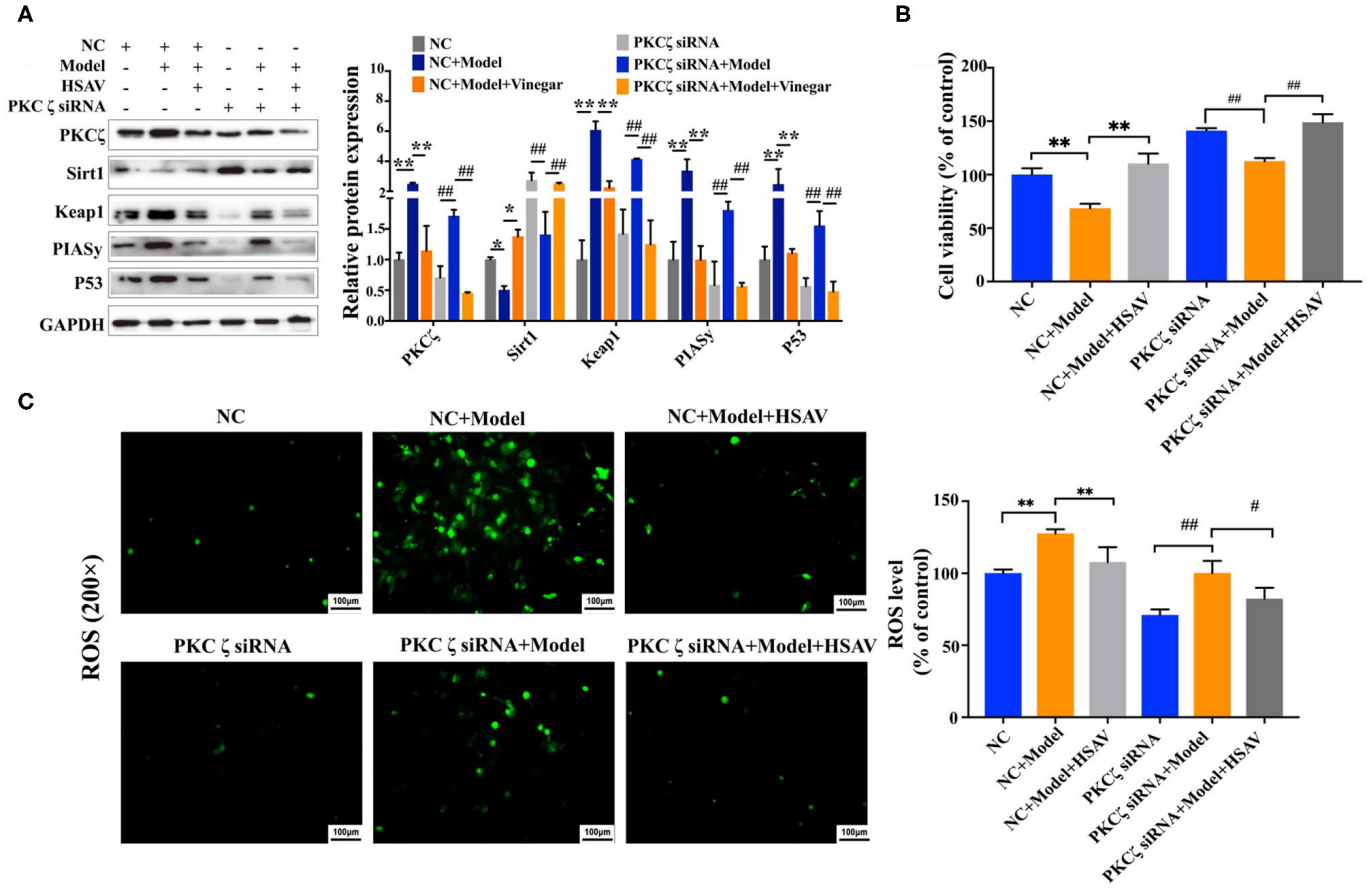
Effect of HSAV on H_2_O_2_ induced injury in HUVECs with PKCζ silencing. **(A)** The expression levels of PKCζ, Sirt1, Keap1, PIASy, and P53 in HUVECs by Western blot assay (*n* = 3); **(B)** cell viabilities of HUVECs (*n* = 6); **(C)** cellular ROS levels (*n* = 3). Data are presented as the mean ± SD.^*^p < 0.05, ^**^p < 0.01, compared with NC+model group; ^#^*p* < 0.05, ^##^*p* < 0.01, compared with PKCζ siRNA+model group.

After PKCζ silencing, the expression of PKCζ in HUVECs was markedly reduced (Figure 7C). Treatment with H_2_O_2_ upregulated the expression levels of PKCζ, Keap1, PIASy, and P53, and downregulated the expression of Sirt1, while HSAV reversed expressions of those proteins compared with PKCζ siRNA+model group (Figure 7C). Consequently, the cell viability was significantly improved and the cellular ROS was reduced by HSAV compared with PKCζ siRNA+model group (Figures 7A,B).

### Effect of Hengshun Aromatic Vinegar on Oxidative Stress and Apoptosis in Human Umbilical Vein Endothelial Cells With Protein Kinase C Zeta Overexpression

**Figure 8:**
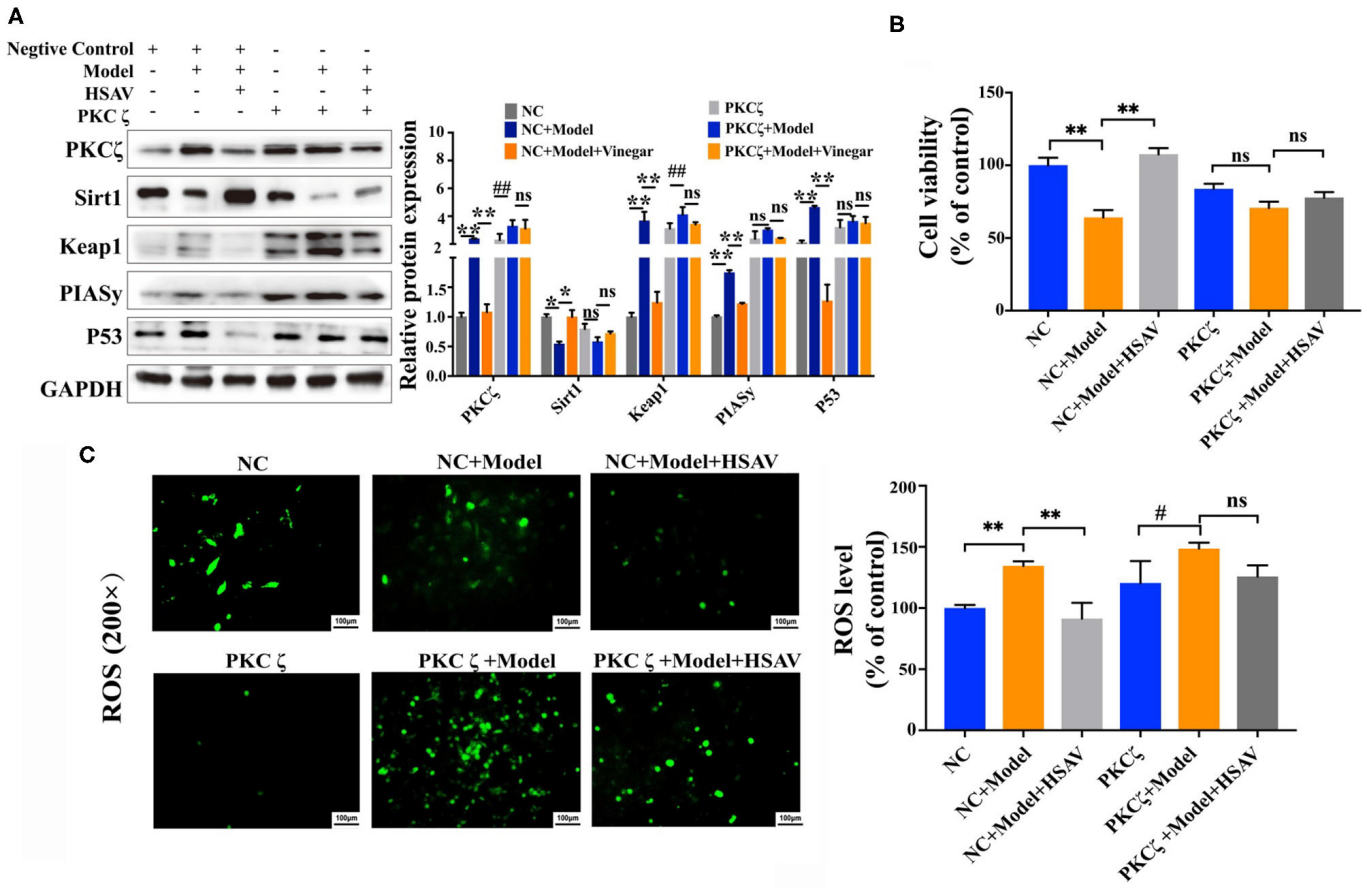
Effect of HSAV on H_2_O_2_ induced injury in HUVECs with PKCζ overexpression. **(A)** The expression levels of PKCζ, Sirt1, Keap1, PIASy, and P53 in HUVECs by Western blot assay (*n* = 3); **(B)** cell viabilities of HUVECs (*n* = 6); **(C)** cellular ROS levels (*n* = 3). Data are presented as the mean ± SD. ^*^p < 0.05, ^**^p < 0.01, compared with NC+model group; ^#^*p* < 0.05, ^##^*p* < 0.01, compared with PKCζ+model group; ns, no significance.

To further determine the effect of HSAV on PKCζ mediated oxidative stress and apoptosis pathway, we overexpressed PKCζ in HUVECs cells. Overexpression of PKCζ blocked the effects of HSAV on the expressions of the PKCζ and downstream proteins including Sirt1, Keap1, PIASy, and P53 (Figure 8A). What is more, overexpression of PKCζ blocked the effect of HSAV on cell viability and intracellular ROS of HUVECs (Figures 8B,C). These findings strongly suggest that protective effects of HSAV on VEI were achieved through regulating PKCζ pathway.

## Discussion

Cardiovascular disease is one of the most important causes of mortality and morbidity in the world. As an important function interface, vascular endothelium plays barrier function, which can maintain normal blood flow and regulate the exchange of substances inside and outside vessels (23–25). Vascular endothelial function can be impaired by ischemia, lipid deposition, and hemodynamic mechanical injury, resulting in VEI (26, 27). VEI is an early event of atherosclerosis, which means that endothelial injury already exists before detectable atherosclerosis (28–31). In this study, VEI model *in vitro* was developed by H_2_O_2_ inducement in HUVECs, and model in rats was established by high-methionine diet, which is more close to human VEI caused by high Hcy.

With the progress in medical technology, therapeutic strategies of diseases have gradually changed from the traditional treatment to comprehensive mode containing prevention, treatment, and rehabilitation. Human beings no longer rely solely on chemical drugs, but pay more attention to prevent diseases. Epidemiological investigations found that food contains many effective ingredients for disease prevention because of their antioxidant properties (32, 33). HSAV is one of the most famous traditional vinegars in China. HSAV is brewed with high-quality glutinous rice as the main raw material through traditional “solid layered” fermentation process, and adopts the typical natural multi-strain mixed fermentation process. Vinegar is rich in lactic acid and amino acids, which can inhibit the formation of lipid peroxidation in aging. It also has features of activity in softening blood vessels and reducing cholesterol, which is beneficial to cardiovascular patients (21, 34). Therefore, we performed a series of analysis to investigate the protective role of HSAV on the VEI both *in vitro* and *in vivo*. HSAV increased cell viability, reduced generation of ROS and inhibited apoptosis in H_2_O_2_-induced HUVECs. HSAV decreased Hcy, ox-LDL, and ET-1 levels, increased NO and eNOS levels, and ameliorated pathological changes in the thoracic aorta in methionine-induced rats. At the same time, HSAV relieved oxidative stress by decreasing MDA levels and increasing SOD, GSH, and GSH-Px levels, and inhibited apoptosis of thoracic aorta cells in rats with VEI.

**Figure 9:**
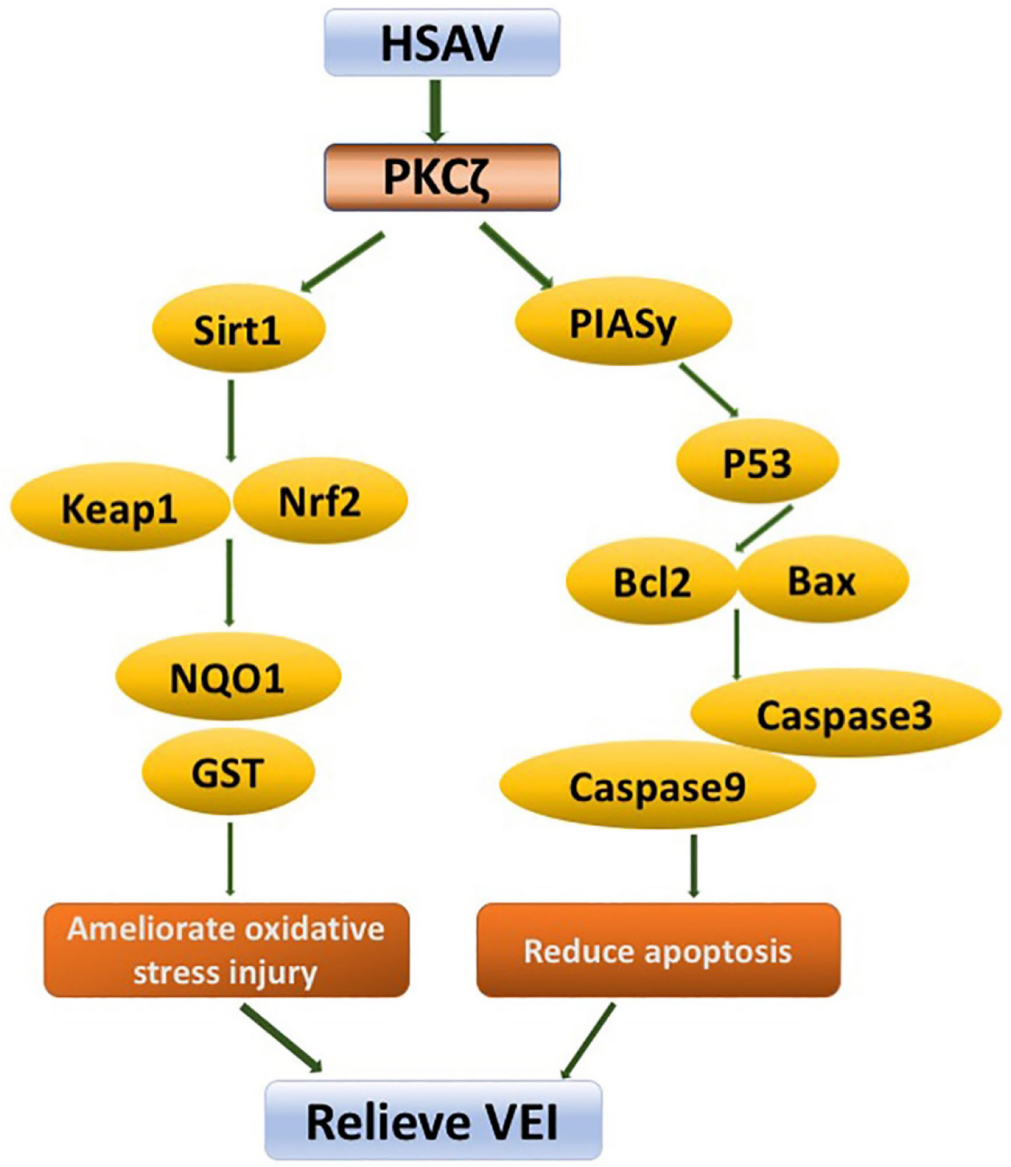
Schematic diagram of molecular mechanism of HSAV against vascular endothelial injury (VEI).

PKC isoenzymes are different in molecular structure, showing tissue-cell specificity in function and activation mechanism (13, 35). More and more experiments have displayed that PKCζ plays an important role in the regulation of intracellular signal transduction induced by various extracellular stimuli (36, 37). In endothelial cells, PKC activation inhibits endothelial NO synthase (eNOS) activity, resulting in decreased endothelial NO release and increased superoxide and hydrogen peroxide production via NADPH oxidase (38, 39). Thus, inhibition of PKC increases the bioavailability of endothelial NO. PKCζ can regulate the activity of Sirt1, which is a deacetylating enzyme of NAD^+^ depletion that has significant antioxidant activity by deacetylating various substrates involved in gene silencing, cell cycle, and aging processes (40). The activation of Sirt1 can significantly improve endothelial function in aged mice (41), decrease the expression of Keap1 that can lead to the dissociation and nuclear translocation of Nrf2, activate Keap1/Nrf2 antioxidant pathway, and thus activate the transcriptions of a variety of antioxidant genes, including NQO1 and GST (42, 43). Therefore, inhibition of PKCζ can decrease the production of ROS and alleviate oxidative stress through regulating Sirt1 pathway. In this study, PKCζ expression was elevated in the model group, which is consistent with the findings in other studies, while HSAV significantly downregulated the expression of PKCζ in injured HUVECs and thoracic aorta tissue of rats with VEI. Furthermore, HSAV significantly upregulated the expressions of Sirt1, Nrf2, NQO1, and GST, and decreased the expression of Keap1 (Figure 9). Consequently, HSAV relieved oxidative stress in HUVECs and rats with VEI by decreasing MDA level and increasing SOD, GSH, and GSH-Px levels.

In addition, activation of PKCζ can increase the binding of PIASy to PKCζ and regulate the activity of PIASy. PIASy inhibits the DNA-binding activity of P53 in nuclear extracts and blocks the ability of P53 to regulate expressions of its target genes, including Bcl2 and BAX (44, 45). It leads to the depolarization of mitochondrial membrane and participates in the process of apoptosis. In this study, HSAV decreased the expression of PKCζ, thereby inhibiting the expressions of apoptosis-related proteins, including PIASy, P53, BAX, Caspase9, and Caspase3, and elevated the apoptosis inhibitory protein Bcl2 (Figure 9), which significantly inhibited apoptosis of HUVECs and thoracic aorta cells of rats with VEI. These results demonstrated that HSAV played a critical role in relieving VEI by ameliorating oxidative stress and inhibiting apoptosis through the PKCζ pathway. Moreover, the findings were also confirmed by knockdown and overexpression of PKCζ.

## Conclusions

This study disclosed that HSAV showed a protective effect on VEI by regulating the PKCζ pathway to improve antioxidant capacity and reduce apoptosis. This study not only provides guidance for the consumption of vinegar in daily life but also promotes the development of dietary supplement for disease prevention.

## Data Availability Statement

The original contributions presented in the study are included in the article/supplementary material, further inquiries can be directed to the corresponding author/s.

## Ethics Statement

The animal study was reviewed and approved by Institutional Ethics Committee of Dalian Medical University.

## Author Contributions

XL and MG designed the experiments, did the experimental part, analyzed the data, and wrote the manuscript. SZ, LY, and BZ performed the animal and cell experiments. YQ and YZ performed the Western blotting assays. YY and LX edited the manuscript. All authors contributed to the article and approved the submitted version.

## Conflict of Interest

XL, SZ, BZ, and YY were employed by Jiangsu Hengshun Vinegar Industry Co., Ltd. The remaining authors declare that the research was conducted in the absence of any commercial or financial relationships that could be construed as a potential conflict of interest.
